# The role of estrogen in cardiac transplantation: mechanistic insights and effects on clinical outcomes

**DOI:** 10.3389/frtra.2025.1734545

**Published:** 2026-01-09

**Authors:** Rosalie Wolff von Gudenberg, Constantin Kupsch, Linda Gilles, Yao Xiao, Catalina Ortiz-Koh, Arjang Ruhparwar, Hao Zhou, Stefan G. Tullius

**Affiliations:** 1 Division of Transplant Surgery, Department of Surgery, Brigham and Women’s Hospital and Harvard Medical School, Boston, MA, United States; 2 Department of Cardiothoracic, Transplantation and Vascular Surgery, Hannover Medical School, Hannover, Germany; 3 Department of General and Visceral Surgery, Medical Center-University of Freiburg, Freiburg, Germany; 4 Department of General, Visceral and Transplant Surgery, Charité, Berlin, Germany

**Keywords:** cardiac allograft vasculopathy, cardiac rejection, estrogen, immunosuppression, primary graft failure

## Abstract

Sex hormones profoundly shape immune responses and influence outcomes after heart transplantation. Estrogen enhances allosensitization and is associated with a higher incidence of acute rejection in female recipients. Beyond its immunological effects, estrogen also modulates the pharmacokinetics and pharmacodynamics of calcineurin inhibitors—particularly cyclosporine A—thereby influencing immunosuppressive efficacy and early graft performance. Donor–recipient sex mismatch further modulates transplant outcomes. Female-to-male transplants in particular exhibit the poorest short- and long-term survival and show increased rates of primary graft failure and cardiac allograft vasculopathy. Mechanistic and experimental data provide a biological basis for these observations: estrogen protects the myocardium against ischemia–reperfusion injury and preserves vascular integrity through both nuclear estrogen receptors and GPER-mediated signaling. Abrupt withdrawal of this estrogen-mediated protection in male recipients of female donor hearts may therefore increase susceptibility to early graft dysfunction and chronic vasculopathy. Integrating sex and hormonal status into transplant medicine—through hormonal profiling, receptor-specific mechanistic studies, and sex-adapted immunosuppressive strategies—may pave the way toward more individualized and effective therapeutic approaches in heart transplantation.

## Introduction

Sex differences in outcomes of heart transplantation have been widely reported. While multiple factors may contribute, accumulating evidence highlights a critical role of sex hormones in shaping immune responses, vascular adaptation, and tissue remodeling following transplantation (1, 2).

Clinically, the impact of biological sex has been observed both, early and late after transplantation. Female recipients—particularly premenopausal women—demonstrate in general higher rates of acute rejections, consistent with their generally more robust immunity. Conversely, male recipients and postmenopausal women may be at lower risk for early rejections but show less favorable long-term outcomes related to comorbidities and adverse vascular remodeling. Sex-related differences also affect post-transplant complications including primary graft failure (PGF) and cardiac allograft vasculopathy (CAV) (3–6).

In general, sex hormones exert profound and pleiotropic effects on immune cells, endothelial function, and cardiomyocytes—all of which are central to graft acceptance or rejection. Estrogen influences the activation and polarization of innate immune cells, context dependent enhances or suppresses adaptive immunity while exerting protective effects against ischemia-reperfusion injury (7).

While estrogens have received substantial attention for their immunomodulatory and cardioprotective effects, testosterone also contributes to sex-specific differences after heart transplantation. In men, testosterone influences hemostasis by enhancing platelet aggregation and coagulation activity (8, 9). Testosterone has also been associated with a less pro-inflammatory immune phenotype, characterized by lower levels of pro-inflammatory cytokines (10–12). These hormonal effects may further shape sex-dependent risks for vascular complications and thrombotic events in the post-transplant setting.

Understanding how sex hormones modulate immune responses and complications following transplantation is thus essential to unravel the biological mechanisms underlying sex-based disparities in heart transplantation potentially paving the way for hormone-informed or sex-adapted immunosuppressive strategies. In this review we focus on the role of estrogens and their effects on alloimmunity and transplant outcomes.

### The role of estrogen in cardiac rejection

The influence of biological sex including the effects of sex hormones on immune responses and transplantation has been in the center of recent investigations.

**Figure 1:**
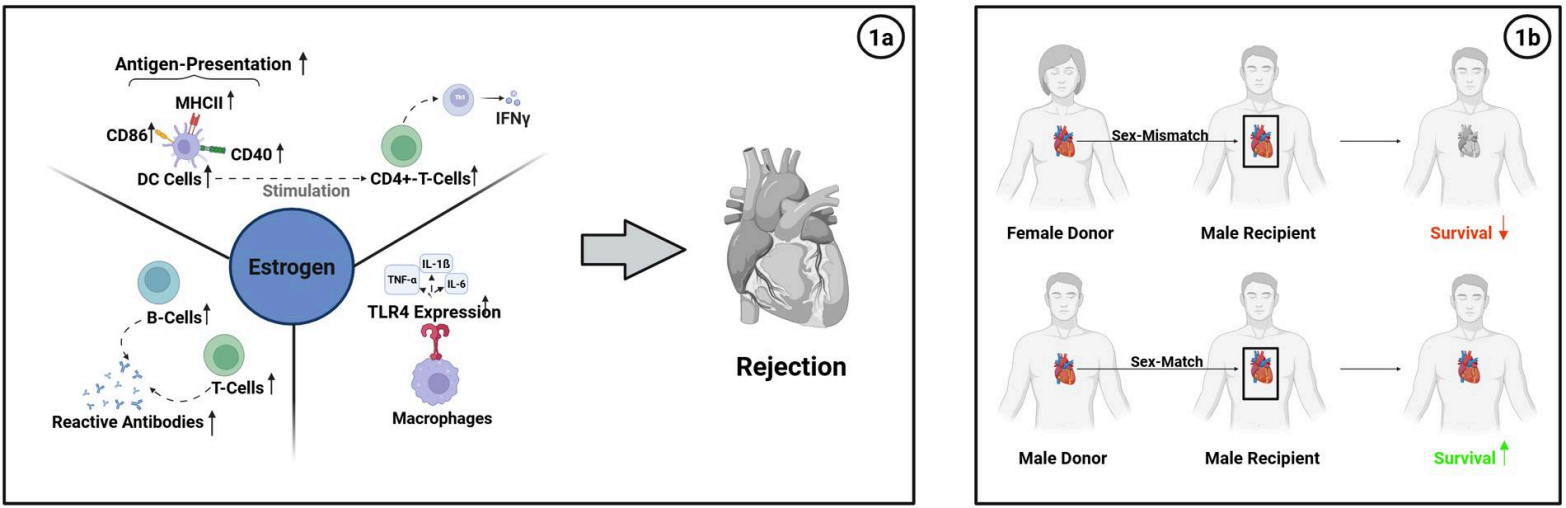
**(a)** impact of estrogen on the alloimmune response after heart transplantation. Estrogen enhances both innate and adaptive immune activation, contributing to higher rejection rates in premenopausal female recipients. **(b)** Survival differences depending on donor-recipient sex combinations. Transplantation of female donor hearts into male recipients (sex-mismatch) is associated with reduced post-transplant survival, whereas male-to-male (sex-match) combinations show improved graft function and survival outcomes.

It has been reported that female recipients of heart transplants have a higher incidence of acute cellular and antibody-mediated rejection post-heart transplant compared to men (13). Of relevance, premenopausal women have a higher frequency of circulating B- and T-lymphocyte counts with augmented immunoglobulin levels when compared to men or postmenopausal women (14) (Figure 1a), in part due to factors that include previous pregnancies, transfusions, and prior transplants (7, 15, 16), reflected clinically by elevated Panel Reactive Antibody (PRA) levels (7, 15, 16).

Importantly, these differences are further amplified by estrogens exerting strong immunostimulatory effects through its receptors expressed on immune cells—including T and B cells, dendritic cells, and macrophages—thereby shaping both innate and adaptive immune responses (7, 15).

Murine studies have shown that estrogens promote the differentiation of bone marrow progenitors into CD11c⁺CD11b⁺ dendritic cells (DCs) with an augmented expression of MHC class II, CD40, and CD86, thus enhancing antigen-presentation (Figure 1a). Estrogens further augment DC-mediated stimulation of CD4⁺ T cells, leading to enhanced T-cell proliferation, migration and Th1 cytokine production including IFN-*γ*, all promoting graft rejection (7, 15–17) (Figure 1a). In turn, inhibition of estrogen signaling through blockade of estrogen receptors on immune cells with the anti-estrogen agent tamoxifen has shown beneficial effects on heart allograft survival in female recipients (18).

Similarly, in other experimental models, estrogen deprivation has been shown to reduce TLR4 (Toll-like Receptor 4) expression, a key regulator of innate immunity, an effect that had been shown to be reversed by 17*β*-estradiol treatment (19). TLR4 activation on macrophages, in turn, triggers the production of proinflammatory cytokines (e.g., TNF-α, IL-1β, IL-6), enhancing T-cell immunity (Figure 1a). Thus, estradiol enhances innate immune activation via TLR4 upregulation, leading to a robust activation of antigen-presenting cells, enhancing T-cell priming, linked to augmented acute rejection rates.

Thus, an augmented allosensitization combined with more robust immune functions driven by estrogens lead to a higher incidence of acute rejections in female heart transplant recipients, with a particular relevance during the first year after transplantation (3).

### Immunosuppression: sex-specific responses to calcineurin inhibitors in heart transplantation

Sex-specific differences in the pharmacokinetics and pharmacodynamics of many immunosuppressants have been demonstrated in organ transplantation.

We have been able to show in a murine transplantation model, that survival differences between male, female, and ovariectomized female recipients were preserved even under immunosuppression with CTLA4-Ig (16). Specifically, graft survival in young female recipients was significantly shorter compared to males, whereas survival in ovariectomized and aged female mice was markedly prolonged relative to young females. These findings highlight that sex hormones can modulate transplant outcomes independently of T-cell–directed immunosuppression, raising the question of how biological sex also affects efficacy and metabolism of established immunosuppressive agents used for heart transplantations.

CNI's including cyclosporine A (CsA) and tacrolimus (TAC) have shown sex-specific differences in pharmacokinetics or pharmacodynamics (20) mainly attributed to sex-specific differences in the CYP3A metabolic pathway (21). Accordingly, in a rat heart transplantation model, female rats under Ciclosporin A have shown a compromised transplant survival with augmented cellular rejection rates.

In support, an additional study demonstrated prolonged cardiac allograft survival in ovariectomized female recipients treated with CsA (18). Notably, administration of estradiol to adult male recipients treated with CsA significantly reduced graft survival, indicating that estradiol may antagonize the immunosuppressive efficacy of CsA. Furthermore, CsA in combination with the anti-estrogen agent tamoxifen significantly improved allograft survival in female recipients and in ovariectomized animals treated with estradiol (18).

Interestingly, CNI-specific effects have been shown in a study comparing CSA with TAC investigating 250 heart transplant patients on either cylosporine (CSA) or tacrolimus (TAC). This study found that Antibody mediated rejection (AMR) was significantly higher in women on CSA but not on TAC (22).

Collectively, these findings suggest that estrogens modulate pharmacokinetics and pharmacodynamics of CNIs, particularly CsA, thereby influencing graft outcomes. Those studies highlight the potential value of sex-specific tailoring of immunosuppressive regimens in heart transplantation. Further studies are required to confirm these observations and to guide evidence-based adjustments in clinical practice (23).

### The effect of sex-mismatches in heart transplantations

#### Impact of donor–recipient sex mismatch on survival

While female pre-menopausal recipients have more frequent acute rejection rates, at least in part related to estrogen-driven immune activation, outcomes after heart transplantation are further shaped by donor–recipient sex mismatch. Indeed, both short- and long-term survival differ significantly when donor and recipient are not sex matched (1, 5, 24–27).

Recent analyses of donor–recipient sex combinations in heart transplantation show consistently that short-term survival is impacted by the interplay between donor and recipient sex, particularly within the first year after transplantation (27). Across multiple studies, male recipients receiving female donor hearts (mR/fD) had the poorest 1-year graft survival and highest early mortality rates (Figure 1b). In a single center, large cohort study, their 1-year survival was ∼79%, compared to ∼84% for male recipients of male donor hearts (mR/mD) (5, 28). Another study analyzing data from 869 adult orthotopic heart transplant recipients between 1980 and 2004, reported a 1-year mortality rate of 24% for mR/fD vs. 13% for the mR/mD constellation, with effects most pronounced in male recipients > than 45 years (28)^,^^3^; a multivariate analysis confirmed female donor sex as an independent risk factor for mortality in this group (odds ratio ≈ 2.3) (28). This trend was also observed long-term (after 5-years) in the mR/fD constellation (26). Conversely, some donor–recipient sex combinations show more favorable short-term outcomes. Female recipients of male donor hearts (fR/mD) often achieve the highest 1-year survival rate suggesting a potential early post-transplant benefit for this combination (24, 29). Aspects in addition to biological sex and hormonal factors such as size discrepancies may also play a role (30).

Beyond overall survival, sex mismatch has also been linked to distinct post-transplant complications including early complications such as primary graft failure (PGF) in addition to long-term complications such as cardiac allograft vasculopathy (CAV). These differences may be linked to estrogens, exerting effects not only via immune cells but also through receptors expressed on endothelial cells, cardiomyocytes, and fibroblasts, affecting immune activation and tissue remodeling (3, 7, 15).

#### Impact of sex-mismatch on primary graft failure

**Figure 2:**
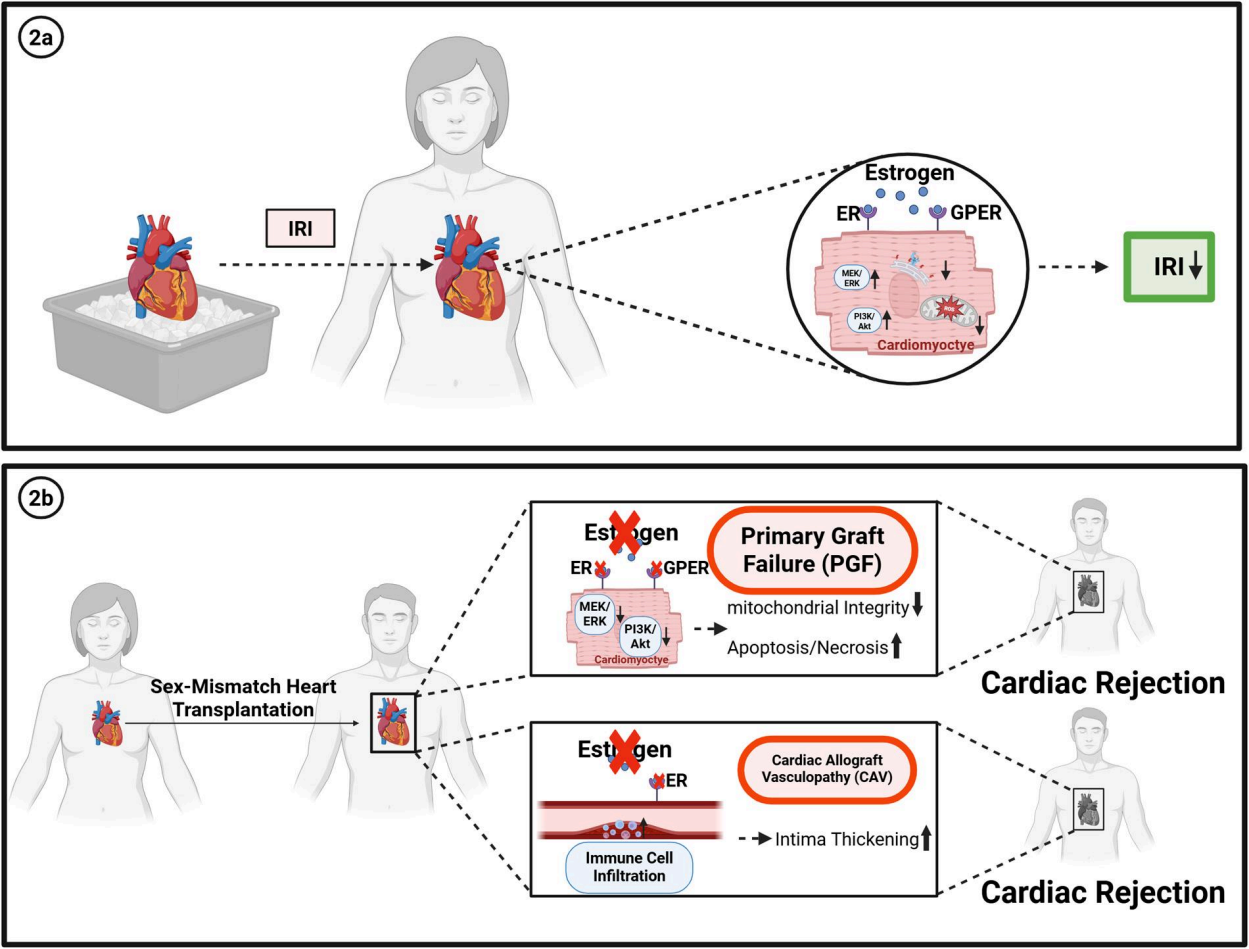
**(a)** protective effects of estrogen on ischemia–reperfusion injury (IRI) after heart transplantation. Estrogen exerts cardioprotective effects through multiple mechanisms: Estrogen reduces reactive oxygen species (ROS) generation after IRI, thereby preserving mitochondrial integrity. Binding to estrogen receptors (ER*α*/ER*β*) and the membrane G protein–coupled estrogen receptor (GPER) activates downstream signaling cascades, including the MEK/ERK and PI3 K/Akt pathways, promoting cell survival and cardioprotection. cEstrogen increases sarcoplasmic reticulum Ca²⁺ ATPase (SERCA2a) expression and reduces endoplasmic reticulum stress, thereby alleviating ischemic injury and improving contractile recovery. **(b)** Potential mechanisms contributing to worse outcomes in sex-mismatched female-to-male heart transplantation.

Primary graft failure (PGF) is characterized by severe myocardial dysfunction within the first 72 h after transplantation, a leading cause of early post-transplant mortality (31, 32). The pathophysiology of PGF is multifactorial, involving donor- and recipient-related factors with ischemia–reperfusion injury (IRI) considered as a central driver (33). During IRI, abrupt restoration of blood flow after ischemia induces oxidative stress, calcium overload, mitochondrial dysfunction, and subsequent cardiomyocyte apoptosis and necrosis, ultimately compromising graft contractility. Notably, PGF has been observed at higher rates in the female-to-male donor/recipient combination (34), suggesting that sex hormones may influence susceptibility to IRI-induced myocardial injury (Figure 2a).

Estrogens have been shown to exert cardioprotective effects against IRI, acting through both classical nuclear estrogen receptors (ER*α* and ER*β*) and the membrane-associated G protein–coupled estrogen receptor (GPER). Activation of ER*α* and ER*β* stimulates pro-survival signaling cascades such as the PI3 K/Akt and ERK1/2 pathways, which inhibit apoptosis and enhance cardiomyocyte viability (35, 36) (Figure 2a).

Those mechanisms are particularly relevant in the context of primary graft failure (PGF), where ischemia–reperfusion–induced mitochondrial injury and cardiomyocyte loss are central drivers of early graft dysfunction. In murine I/R models, administration of an ER*β* agonist or estradiol (E2) reduced necrosis and apoptosis, as evidenced by decreased LDH release and fewer TUNEL-positive cardiomyocytes. At the molecular level, the anti-apoptotic protein Bcl2 and the mitochondrial protein acetyl-coenzyme A acyltransferase 2 (ACAA2) (37) were increased in mitochondrial fractions of ER*β*A- and E2-treated hearts. Moreover, mitochondrial integrity was preserved, reflected by higher levels of the mitochondrial translocase TIM23 in estrogen treated groups. Functionally, these molecular effects translated into improved left ventricular developed pressure and recovery of contractile performance after reperfusion (38).

Additional studies using hypoxia–reoxygenation models in rat cardiomyocytes further support these findings. Pre-ischemic treatment with E2 enhanced expression of the sarcoplasmic reticulum Ca^2^⁺ ATPase pump (SERCA2a) while reducing endoplasmic reticulum (ER) stress–related protein levels, thereby alleviating myocardial injury (39) (Figure 2a). Consistently, estrogen deprivation through bilateral ovariectomy reduced SERCA2a levels and exacerbated myocardial infarction size, apoptosis, troponin I release, and morphological injury after I/R. These results underscore the pivotal role of estrogen in mitigating myocardial damage by maintaining calcium handling and suppressing ER stress (39).

Similarly, the G protein–coupled estrogen receptor (GPER) has emerged as an important mediator of estrogen signaling (40). Unlike the classical nuclear receptors ER*α* and ER*β*, GPER is a transmembrane receptor activating rapid, non-genomic pathways upon estrogen binding. Activation of GPER triggers downstream signaling cascades including the MEK/ERK (MAPK kinase) and PI3 K/Akt pathways, which have been linked to cell survival and cardioprotection (41). Accordingly, *in-vivo* studies of rat hearts that were subjected to ischemia followed by reperfusion with the selective GPER agonist G1, have shown protection against ischemia/reperfusion injury (41, 42).

Additional experiments demonstrated that post-ischemic administration of estradiol also conferred protection against IRI by preserving mitochondrial structural integrity, reducing ROS generation while stabilizing the mitochondrial membrane potential (43, 44) (Figure 2a). More specifically, estrogen increased the calcium threshold required for mitochondrial permeability transition pore (mPTP) opening through the activation of the MEK/ERK/GSK-3β axis, thereby preventing premature pore opening and maintaining mitochondrial function subsequent to reperfusion. In addition, estradiol attenuated excessive mitophagy via modulation of the PINK1/Parkin pathway involving LC3I, LC3II, and p62 proteins (41). Functionally, these protective mechanisms translated into reduced post-ischemic dysfunction and a significant decrease in infarct size following I/R.

Taken together, these findings indicate that estrogen protects the myocardium against ischemia–reperfusion injury through both nuclear ERs and GPER-mediated signaling, thereby preserving mitochondrial integrity, reducing apoptosis with an improved functional recovery. As IRI represents a key determinant of primary graft failure (PGF), these mechanisms provide a biological underpinning for the observed sex differences in transplant outcomes. In particular, the absence or reduction of estrogenic signaling in female to male recipients may exacerbate IRI-induced myocardial damage, thereby increasing susceptibility to PGF and contributing to the higher early post-transplant mortality observed in these groups (Figure 2b).

#### Impact of sex-mismatch on cardiac allograft vasculopathy

Allograft vasculopathy (CAV) is the hallmark of chronic rejection after heart transplantation histopathological characterized by (i) loose connective tissue with inflammatory cells, (ii) lesions with smooth muscle cells, and (iii) fibrotic lesions (45). The most prominent feature distinguishing CAV from atherosclerosis is fibromuscular hyperplasia of the intima**.**

According to the Registry of the International Society for Heart and Lung Transplantation (46), CAV is the leading cause of death between 1 and 3 years after transplantation (4). Progressive endothelial dysfunction and intimal thickening eventually result in vascular fibrosis, microvascular dysfunction, and graft failure, all limiting long-term graft survival (4, 47). Both immunologic and non-immunologic risk factors contribute to the development of CAV, with biological sex playing an important role.

Male recipient sex has been independently associated with higher CAV rates (6). In sex-mismatched transplantations, male recipients of female donor hearts exhibited significantly more advanced intimal thickening, as demonstrated by intravascular ultrasound by one year post-transplantation (47–49) (Figure 2b). In contrast female recipients receiving a male allograft developed a less severe thickening of the intima (49, 50).

While the underlying mechanisms remain under investigation, accumulating evidence suggests that the loss of the estrogen-protective environment of the female heart may represent a key factor for the higher CAV rates observed in female to male transplants (49, 51). Estrogen has been shown to exert protective effects on the vasculature by promoting endothelial regeneration and limiting neointimal formation, thereby potentially mitigating CAV progression.

Outside of transplantation, observational studies suggest that estrogen replacement therapy (ERT) impacts the risk of coronary heart disease subsequent to postmenopause (52, 53). Consistently, studies of postmenopausal women demonstrated that hormone replacement therapy delayed thickening of the atheromatous intima layers in carotid and femoral arteries (54, 55).

Experimental models elucidate some of the mechanisms underlying the protective effects of estrogen on the vasculature. In a rabbit cardiac allograft model, estradiol administration abolished MHC class II expression in graft coronary arteries, markedly reduced macrophage and T-cell infiltration, resulted in a 60% reduction in myointimal thickening, thus highlighting the capacity of estrogens to attenuate graft vasculopathy (56). In support, ovariectomized rats subjected to balloon-induced carotid injury exhibited dose-dependent acceleration of re-endothelialization and enhanced nitric oxide production under estradiol replacement, linked to an ameliorated neointimal proliferation (57, 58). Of additional relevance, in female cynomolgus macaques on an atherogenic diet, estradiol administration has been shown to reduce plaque area by 50% (59). Additional mechanistic insights also point towards direct vascular effects: in ER*α*-deficient mice, estrogen continued to inhibit injury-induced smooth muscle proliferation, suggesting receptor-independent pathways (60).

Likewise, in cuff-induced femoral artery injury, estrogen suppressed vascular smooth muscle cell (VSMC) proliferation and migration (61), while in cultured VSMCs estradiol directly inhibited DNA synthesis. Additional studies in rats confirmed that these antiproliferative effects are at least partly mediated via nitric oxide–cGMP–cAMP signaling (62).

Taken together, these findings demonstrate that estrogen protects against vascular remodeling by promoting endothelial repair, enhancing endothelial function, inhibiting smooth muscle cell proliferation and migration, and reducing immune cell infiltration. Clinically, these effects may help to explain the higher incidence of CAV in male recipients as well as the inferior outcomes observed in male recipients of female donor hearts. In female to male donor/recipient combination, the transplanted organ is suddenly deprived of estrogen-mediated protection, predisposing to endothelial injury, immune activation, neointimal thickening, and fibrosis all hallmarks of CAV pathogenesis.

### Outlook and future perspectives

The growing recognition of sex-specific differences in heart transplantation highlights the urgent need to incorporate biological sex and hormonal status into clinical decision-making. Future studies may aim to elucidate the precise mechanisms by which estrogen modulates immune cell function, endothelial integrity, and tissue repair in the context of cardiac transplantation. Notably, the dual role of estrogen remains an unresolved challenge in transplantation research (63). While estrogens confer tissue protection by mitigating ischemia–reperfusion injury through activation of pro-survival and anti-oxidative pathways, they simultaneously enhance immune activation by promoting dendritic cell maturation, T-cell proliferation, and antibody production. This context-dependent duality—protective at the graft tissue level yet immunostimulatory at the systemic level—may help explain the paradox of improved ischemic tolerance but increased rejection rates in young female recipients. Understanding how receptor-specific signaling and hormonal milieu determine these opposing outcomes will be crucial for therapeutic translation.

As estradiol concentrations vary substantially interindividually, across menstrual cycle phases, and physiological stress, measuring circulating estrogen levels alone is unlikely to accurately reflect individual risk. Rather than absolute hormone levels, the overall hormonal milieu — including menopausal status, age, and pregnancy history — is more likely to support a risk assessment, particularly in sex-mismatched graft settings.

Thus, prospective studies with pre-defined time points for hormone measurements, interpreted in the context of these broader determinants, together with detailed immunophenotyping are needed. Such an approach may help clarify how dynamic hormonal patterns relate to graft survival, rejection risk, and cardiac performance. Moreover, there is a rationale to explore sex-adapted immunosuppressive strategies (23, 64, 65). Understanding how to harness the protective effects of estrogen, or compensate for its absence in male recipients may improve both graft and patient survival. Ultimately, integrating sex-based immunobiology into transplant medicine may pave the way for more precise, individualized, and effective therapies in the future.
